# Treatment of Two-Phase Olive Mill Wastewater and Recovery of Phenolic Compounds Using Membrane Technology

**DOI:** 10.3390/membranes9020027

**Published:** 2019-02-05

**Authors:** Varvara Sygouni, Alexis G. Pantziaros, Iakovos C. Iakovides, Evangelia Sfetsa, Polychronia I. Bogdou, Emilia A. Christoforou, Christakis A. Paraskeva

**Affiliations:** 1 Department of Chemical Engineering, University of Patras, GR-26504 Patras, Greece; sygouni@upatras.gr (V.S.); alexispantzi@gmail.com (A.G.P.); iakovidis.iakovos@ucy.ac.cy (I.C.I.); sfetsa.euaggelia@gmail.com (E.S.); polybogdou@yahoo.gr (P.I.B.); emily.chris@hotmail.com (E.A.C.); 2 Foundation for Research and Technology Hellas, Institute of Chemical Engineering Science, Stadiou Str., Platani, GR-26504 Patras, Greece

**Keywords:** phenols, extraction, membrane filtration, two phase pomace, olive mill solid waste

## Abstract

The semi-solid wastes (pomace or alperujo) produced in the two-phase olive oil extraction process contains extremely high organic load and phenolic substances. Efficient treatment of such kinds of wastes using membrane filtration, should be sought to reduce the hazardous effects to the environment. On the other hand, phenolic compounds can be isolated and purified up to a level of commercial exploitation using the membrane technology. Firstly, the extraction process with mixtures of water and ethanol was optimized by testing extraction parameters (e.g., solvent’s mixture, duration, and temperature) at laboratory scale. Next, extraction was conducted using larger volumes and the treatment was continued in a pilot membrane filtration system, consisted of ultrafiltration (UF), nanofiltration (NF) and reverse osmosis (RO) membranes. The extracted solution from the olive oil pomace was fed to the pilot membrane filtration system, where all fat, lipids, and solids were removed while the phenolic compounds were concentrated in the retentate streams of NF and/or RO. Total phenolic content (TPC) at the RO’s concentrate stream was 225 mg/L and at the final effluent was lower than 10 mg/lt. The chemical oxygen demand (COD) value at the final effluent was much lower (~280 mg/L) than in the feed stream (>32,000 mg/L).

## 1. Introduction

The main products from olive trees are table olives and olive oil, which are essential components for a healthy nutrition and are related to the Mediterranean dietary. Among the compounds that make olive oil an important ingredient in a human’s diet, are the contained polyphenol compounds, which show antioxidant activity. The most important phenols present in olive oil are tyrosol, oleuropein, caffeic acid, vanillic acid, and hydroxytyrosol [1,2].

Olive oil extraction processes have been improved during the centuries aiming to increase olive oil production and to improve its quality. During the production of oil, a large amount of waste is produced which is phytotoxic due to its high content in fat, lipids, and polyphenols [3]. Nowadays, the most used types of olive oil mills are the two- and the three-phase decanter systems. An overview of the olive mill wastes, and their valorization methods is given in [4,5] while in [6] a summary of the results achieved in composting experiments of two- or three-phase olive mill wastes is presented.

The two-phase system was introduced in the early 1990s and it was characterized as ecological because it produces fewer amounts of liquid wastes compared to the three-phase system [7] where warm water is used to enhance the extraction of olive oil from the pulp of olive fruits. Moreover, it is less complicated, more reliable, and less expensive than the three-phase one, with significantly lower energy and water consumptions. The two-phase systems are already widely used in Spain, whereas their use is always increasing in other Mediterranean countries such as in Italy, Greece, and Portugal. During the olive oil production using the two-phase system, a semi-solid waste (65% moisture) is produced which is called olive mill solid waste (OMSW) or “two-phase olive pomace” or “alperujo” [8]. On the other hand, the treatment of the wastes from the two-phase systems presents great difficulties caused by the moisture (65% w/w) and carbohydrate concentrations that characterize this type of wastes [9]. As in the case of the wastewater produced from three phase systems (Olive Mill Wastewater, OMW), the OMSW needs to be treated properly due to its high concentration in solid, fat, lipids, carbohydrates, and polyphenols. The classic aerobic or anaerobic methods for the treatment of heavy organic wastewaters are limited by the heavy organic load in terms of fat, lipids, and phenolic compounds [10,11,12] which prohibit microbial growth. OMSW treatment, with parallel recovery of specific byproducts of high added value, such as polyphenols, is of high interest for all Mediterranean countries. Thus, other physicochemical methods are currently being involved for the treatment of agro-industrial wastewaters [13]. Membrane filtration is among the widely used physicochemical methods for the treatment of olive mill wastewaters (OMW) [3,14,15,16,17,18,19]. A recent review paper [20] describes the use of membranes (UF, NF, and RO), as well as membrane bioreactors, vacuum distillation, and osmotic distillation for the effective treatment of Olive Mill Wastewaters. In Paraskeva et al. [15], OMW produced by the three-phase system was treated using a pilot scale set-up consisting of UF, NF, and RO membranes, whereas several operational parameters were investigated for the optimum operation of membranes. A pre-treatment step of OMW filtering was performed in order to reduce membrane fouling. The final permeate (~70%) (final effluent) of the initial volume was found to meet the EU and national regulations, in order to be discharged at the environment without environmental risk or to be used at irrigation systems for water economy. The concentrates obtained from the NF unit of the pilot scale membrane modules were tested as ecological herbicides in a series of experiments with different herbs and along with the possibility of water reuse, the cost for the OMW treatment could be balanced [21]. The membrane unit set-up was combined also with other techniques such as the anaerobic digestion of the OMW using a periodic anaerobic baffled reactor where the decrease of hydraulic retention time enhanced the membrane system retention of the chemical oxygen demand (COD) [22]. Based on a recent literature survey, the most effective methods for OMW purification are membrane filtration, coagulation/flocculation, anaerobic digestion, and Fenton oxidation [13]. An ultrafiltration membrane system, solar photo Fenton oxidation, and UF and NF module membrane systems were compared for their OMW treatment efficiency while cost and SWOT analysis showed that the use of a photo Fenton oxidation method combined with a needed pre-treatment step as well as membrane system filtration, are of the most efficient methods [23]. Membrane filtration was also used for the isolation of organic compounds of high added value from agro-industrial solid wastes and wastewaters coming from olive oil mills and wineries which do not accomplish the criteria for commercial use [24]. In this direction, several techniques or the combination of techniques have been developed for the simultaneous purification of OMW and the recovery of high added value by-products such as the use of UF, NF. and RO membrane modules, use of resins, and cooling crystallization [25,26]. In the case of OMW high phenolic content was recovered using the UF, NF, and RO membrane module followed by rotary evaporation [26,27,28,29].

In the present study, the already tested methods for the treatment of three phase olive mill waste are used for the treatment of two-phase semi-solid waste (pomace) and are completed by an extra step for the extraction of organics and phenolics from the matrix of solids. While in other works [30,31] these extracted liquids are considered as olive pomace leachates that need effective treatment before their final disposal to aqueous receptors, the present study focusses on the optimization of the extraction of olive pomace leachates in order compounds that are included in those leachates to be evaluated as sources of antioxidants. Methods for the extraction of phenolics from olive pomace are reported in the literature [32,33] where emphasis is given to the extraction of organics from the organic matrix of olive pomace. Araujo et al. [33] tabulated the methods for phenolic compounds extraction based on the type of chemical agent used. Most of the reported studies [34,35,36,37,38] referred to excellent phenolic extraction using mixtures of methanol and water in a volume analogy of 80/20, in the presence of an acid as a catalyst (HCl or H_2_SO_4_) and an n-hexane to remove the lipids. Other research works [39,40] reported extraction with EtOH/H_2_O (80:20 v/v) and at a solvent to sample ratio of 5:1, at low pH values (pH = 2). In the present work, laboratory experiments were performed in order to examine the separation and recovery of the phenolic compounds through extraction of organics using only agents that are safe for food chain, that is water and ethanol only. In the beginning, a parametric investigation aiming at the enhanced phenolic recovery through the extraction experiments was performed. The investigated parameters were: the type of solvent used during the extraction, the addition of small quantities of HCl solution during the extraction, the extraction temperature, the repeatability of extraction, the stirring rate, and the time of extraction. At a second stage, the semi-solid waste was treated, and the phenolic content was isolated using a pilot scale of membranes with larger volumes of extracted solution (120 L). The first membrane in the proposed scheme consisted of an ultrafiltration (UF) module, where all suspended solids, fat, lipids, and high molecular organic molecules were removed. The second membrane was nanofiltration (NF) module, where most of the organics and the total phenolic content (TPC) were isolated. The third unit of reverse osmosis (RO) membrane was used for the tertiary treatment of the permeate stream of NF membrane. The final permeate effluent was almost clean water with limited concentrations of phenolics and organics (in COD values).

## 2. Materials and Methods

### 2.1. Olive Mill Wastewater

The OMW was collected from two different olive mills in Patras (region Achaia, Greece). Both olive mills are using two-phase extraction systems. The first sample was collected during November and the second one on December 2016. The kind of used olives for oil extraction was “Koroneiki” (Olea Europea) which is the most commonly cultivated olive tree variety in the region. The wastewater, after the collection, was separated into smaller vessels and stored at −20 °C to prevent any alteration of its characteristics. The first OMW sample was used for the extraction experiments while the second one was used for the experimental series at the pilot scale.

### 2.2. Extraction Experiments

The extraction laboratory experiments were carried out using a jar test apparatus (Flocculator “FLOC-6” RAYPA). Six beakers of 600 mL were employed, and the extraction took place under mechanical stirring. Next, the extracted solution was separated from the solid matrix using Whatman glass microfiber filters of 1.6 μm pore diameter. In order to obtain the optimum conditions for TPC recovery, a parametric study was performed. Firstly, the optimum type of solvent for extraction was investigated. Water and mixtures of water and organic solvent (95% pure ethanol) were tested at five different concentrations: pure water, 25% ethanol in water, 50% ethanol in water, 75% ethanol in water and 100% ethanol. For the first extraction experiments, 20 g of semi-solid waste were suspended in a solution of 100 mL of solvent, at 25 °C, under stirring (100 rpm, 1 h). Next, all experimental series were performed using two types of solvents: (i) pure water and (ii) a mixture of 50% ethanol and 50% of distilled water (hereinafter 50% E-50% W). The parametric study included investigation of: the optimum solvent; the optimum quantity of semi-solid waste that should be used in each experiment; the optimum dose of HCl solution (1N), that might enhanced hydrolysis of the organics in simple compounds [33]; the temperature; the maximum recovered quantities of phenols (iteration of the extraction with the same solids); the stirring rate; the duration of the extraction time. The required quantity of semi-solid waste that should be used in order to recover the maximum TPC quantity was investigated by performing experiments at 25 °C and 100 rpm of stirring rate, using 100 mL of two different solvents: (a) pure water and (b) 50% E-50% W. The effect of HCl was investigated using 40 g of solid olive oil mill waste and the two different solvents of 100mL for each extraction set. The temperature effect on the extraction of phenolics and carbohydrates was studied in a series of experiments performed with 40 g of solid waste suspended in 100 mL of the two types of solvents where the temperature varied between (10–60) °C. The stirring was constant at a rate of 100 rpm for 1 h. The effect of stirring rate on the extraction of phenols was investigated using 40 g of solid waste for the extraction of organics in a volume of 100 mL of the two solvents. The extraction lasted 1 h, at 25 °C and the stirring was varied at rates of 0, 50, 100, 150, and 250 rpm.

### 2.3. Membrane Units

A pilot scale of membranes [15,21] was used in cross-flow mode and a batch operation were employed. UF unit was ceramic (Zirconia), of 0.36 m^2^ filtration area and 100 nm pore size, with maximum flow up to 9 m^3^/h, maximum pressure up to 9 bars and maximum temperature up to 70 °C. The NF unit was spiral wound (advanced polyamide) with Molecular Weight Cut-Off (MWCO) of 800 Dalton whereas the RO spiral wound membrane was designed to reject the 99% rejection of monovalent ions (NaCl). NF and RO consisted of three layers composite non-cellulosic membranes, which can be used for pressures up to 68 bars and temperature up to 50 °C. All membranes and the pilot plant units were supplied by HAR SpA, Milan, Italy.

The feed tanks of UF and NF units were of 180 L volume and 100 L respectively. During cross flow filtration the concentrated stream was recycled in the corresponding feed tank and the permeate stream was collected in a separate tank until the end. At the end of each experiment, the membranes were cleaned using a NaOH (1N) solution for 30 min and finally using water until neutral values of pH. In the case of the polymeric membrane, bisulfite solution was circulated for 20 min for sterilization.

Trans membrane pressures (TMP) were selected according to the directions of the manufacturing company (HAR SpA, Milan, Italy) and according to the experience of the technical personnel of the group. TMP for UF was 2 bars for NF 10 bars and for RO was 30 bars. Due to the fouling the flowrate was decreased to the half of the initial flowrate.

### 2.4. Total Phenolic Content Measurement

Total phenolic content (TPC) was measured using the Folin-Ciocalteu method, as described in [29]. This method detects the hydroxyphenyl groups which are present in a solution. The phenolic compounds can reduce phosphomolybdic acid and phosphotungstic acid compounds which are contained in the Folin-Ciocalteu reagent. The blue chromophore produced by the reaction, is consisted of a complex which has a maximum absorption depending on the TPC and the alkaline solution. The TPC concentration in gallic acid equivalents is determined by the absorbance value of the compounds obtained using a photometer at 760 nm and a standard concentration versus absorbance curve for known TPC concentrations.

### 2.5. Total Carbohydrates Measurement

The determination of carbohydrates was based on the formation of coffee-violet (for hexoses) or brown-green complexes (for pentoses) between the polyaromatic reagent compounds and the sugar molecules. The method is summarized in [29] along with all recipes and techniques needed for the physicochemical characterization of all streams. It involves a reaction of carbohydrates with L-tryptophan in the presence of borate and concentrated sulfuric acid for 20 min in a boiling water bath and spectrophotometry at 525 nm.

The carbohydrate concentration is calculated either using a standard optical absorption curve, based on the concentration of standard glucose solutions, or by direct measurement of the concentration using the appropriate colorimeter. For the first method, 5 glucose solutions of varying concentrations between 5 and 90 mg/L are prepared. The carbohydrates concentration of each sample is expressed in glucose equivalents. Tryptophan has been shown to produce derivatives of similar absorption intensity with different monosaccharides.

### 2.6. Pilot Scale Experiments

**Figure 1:**
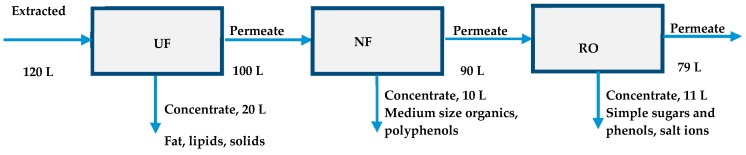
Schematic diagram of the feed, permeate and concentrate streams during the pilot scale experiment. UF: ultrafiltration; NF: nanofiltration; RO: reverse osmosis.

Pilot scale experiments were performed using the pilot plant of UF, NF and RO membranes described previously. Six kilograms of solid waste were used to extract a solution of 120 L based on most of the parameters obtained from the batch extraction process described above. The extracted solution (raw) was sieved carefully with stainless steel sieves with different sizes of pores, the smallest being the one with pore openings of 125 μm. The sieved solution, free of large suspended particles, was introduced into the membrane scheme (Figure 1), however, before the initiation of the experiment this solution was mixed with the water pre-existed in the tubing system (feed). Thus, the concentration of all compounds was reduced by a factor of 5–10% because of the mixing of the filtered raw solution with the existing water. The volume balances in each membrane modules are shown in Figure 1. The permeate stream from UF was used as feed for the NF module while the permeate stream of NF was fed to RO module. What is important is the final treated volume (~80 L) which was almost free of organics and this stream can be considered as appropriate for irrigation or recycling in the premises of the olive mill factory. As for the concentrates, small particles (with size less than 125 μm), fat, lipids, and large molecules with high molecular size were isolated in the concentrate stream of ultrafiltration (20 L). Other organics with intermediate sizes are removed in the concentrate stream of NF (10 L) and all remaining organics were retained in the RO concentrate stream (11 L).

## 3. Results and Discussion

### 3.1. Parametric Investigation of the Extraction Procedure

In the first part of the present work, a parametric investigation was performed in order to find the optimum conditions for the extraction of the maximum quantity of TPC (total phenolic content) from the two-phase olive mill semi solid waste (OMSW or ‘pomace’). In the beginning, the effect of the type of the solvent used during the extraction was investigated. Pure water or ethanol or mixtures of ethanol and water at different percentages were tested. The maximum possible extraction of phenols from the semi-solid pomace was obtained in a series of experiments with different percentages of solid and solvent (g pomaces/L of solvent). The addition of HCl solution during the extraction was also tested in order to study the effect of possible further hydrolysis of organics and of the phenolic compounds. Other important parameters that were tested were the temperature during the extraction, the rate of stirring, and the extraction time. The extraction of phenolics from a certain amount of semi solid samples was repeated in some experiments with fresh solvent in order to find the maximum available quantity of phenolic content that can be extracted from the semi-solid waste.

#### 3.1.1. Effect of the Solvent

Water, ethanol, and mixtures of ethanol/water were used as solvents because these two solvents are readily acceptable in food industry. Methanol was reported also as an excellent solvent [34,35,36,37,38] but it has been accused to cause harm to human health and thus it was avoided.

**Figure 2:**
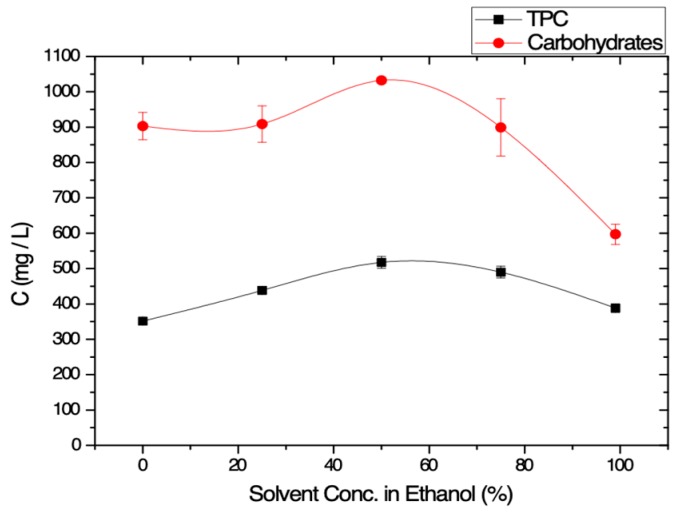
Total phenolic content (TPC) and carbohydrates concentrations obtained after the extraction experiments using solvent of varying concentration in ethanol (95% purity).

Both TPC and carbohydrates concentration values were increased as the fraction of ethanol in water increases until 50% ethanol in water (Figure 2). Japon-Lujan and Luque De Castro [39] reported as best extraction conditions a ratio of ethanol to water equal to 80:20 using a laboratory-made extractor. This difference (EtOH: H_2_O = 80:20) with the present work (EtOH:H_2_O = 50:50) shall be addressed to the fact that in [39], superheated liquid extraction was performed, while in the present work, almost all experiments were performed at ambient temperatures. For higher concentration values of ethanol in water, TPC and carbohydrates concentration values were decreased (Figure 2). Thus, the mixture 50% E-50% W was chosen as the optimum solvent. In all solvent cases, as in all fruits, the concentration of carbohydrates is much higher than the TPC.

#### 3.1.2. Maximum Quantity of Solids Per Solvent

**Figure 3:**
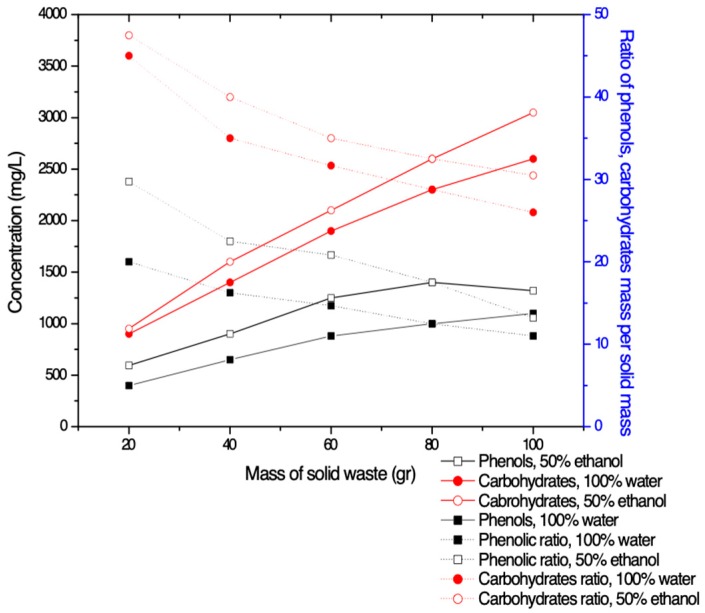
Concentration of TPC and carbohydrates as a function of the mass of solid waste (solid lines), and ratio of TPC and carbohydrates mass per mass of solid (dot lines).

The TPC and carbohydrates recovery using two different solvents: (a) pure water and (b) EtOH/Water: 50% Eth-50% W s a function of the quantity of semisolid waste are summarized in Figure 3. Both TPC and carbohydrates concentration are increased as the amount of used semi-solid material is increased for both solvents, as expected (solid lines).

Figure 3 confirms the remark obtained in Figure 2, that the use of the mixed solvent (50% E-50% W) enhanced the extraction of organics. However, what is important in this case, is the extracted quantity of organics per gram of solid wastes. Dot lines, which are referred to the right axis of Figure 3, suggest that the most effective extraction was obtained when only 20 or even 40 g of solid was diluted in the volume of 100 mL of the solvent. It seems for large mass values of the used solid wastes, the extracted solution is saturated with organics and there is a possibility of phenols or carbohydrates to remain in the mass of the solid waste. From the present investigation the concentration which seems to be optimum for recovering of significant amounts of TPC, and relatively less amount of carbohydrates is the 20 g of waste per 100mL of solvent. However, the value of 40 g was chosen in order a larger quantity of phenols or carbohydrates to be accumulated in the solution that will be further treated with membranes and to isolate larger quantity of phenols. This agrees with the work reported in Lafka et al. [40] where the best extraction method was delivered with EtOH mixtures and a solvent to sample (solid) ratio of 5:1.

#### 3.1.3. Effect of HCl

**Figure 4:**
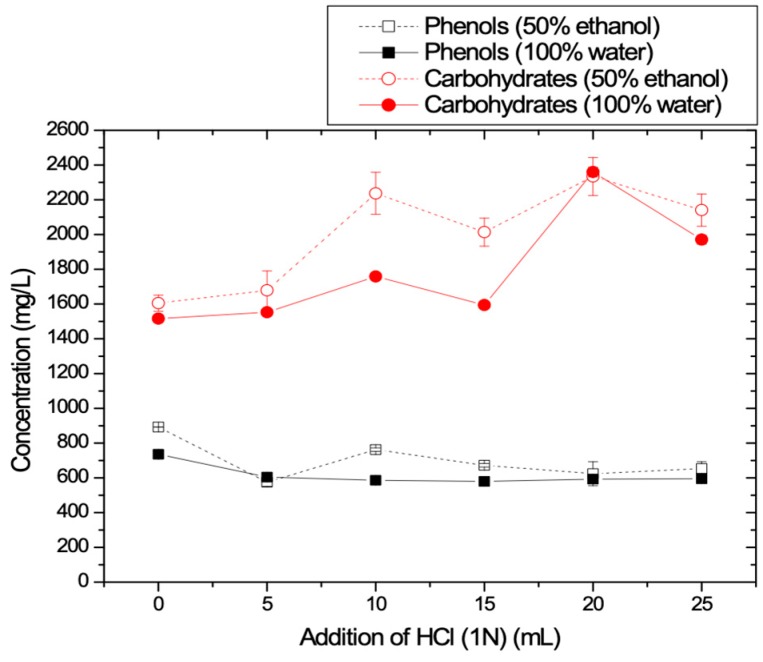
TPC and carbohydrates concentrations as a function of added HCl (1N).

HCl or acid environment (pH~2) has been reported in the literature as a reagent that might help hydrolysis of organics and aiming to improve the recovery of phenolics [8,34,35,40]. The effect of HCl addition on TPC and carbohydrates recovery for each type of solvents is summarized in Figure 4. It is shown that TPC concentration values for both solvents was slightly decreased upon the addition of HCl. On the contrary, the obtained carbohydrates concentration values, were found to be increased with the addition of HCl. Figure 4 suggests that HCl could be avoided because it does not help the enhancement of the recovery of the phenolic compounds despite the fact the literature suggests that HCl increases the rate of organic hydrolysis [33].

#### 3.1.4. Temperature Effect

**Figure 5:**
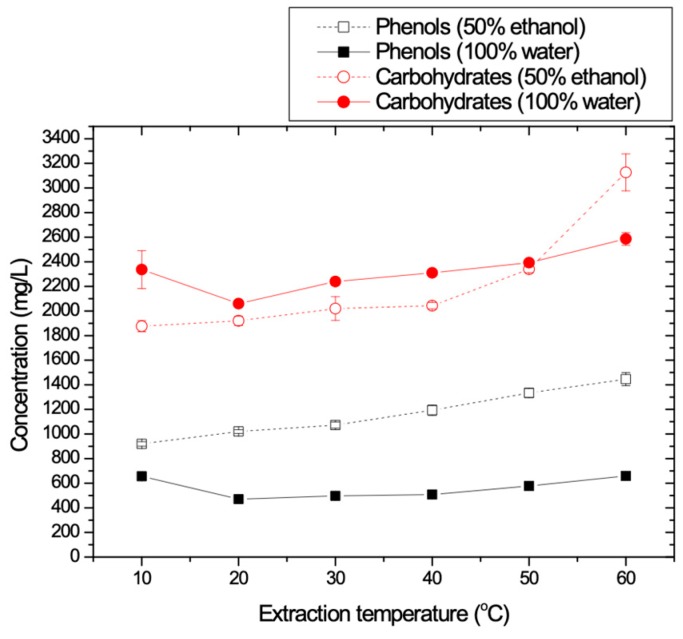
TPC and carbohydrates concentrations as a function of the extraction temperature.

The effect of the extraction temperature on TPC and carbohydrates recovery is depicted in Figure 5. The obtained results show that there is a significant increase in TPC values when the temperature was increased up to 60 °C when the second solvent was used (50% E-50% W). The extraction of TPC with pure water as solvent gave much lower values of TPC for all tested temperature values.

However, carbohydrates concentration values were higher when the extraction was performed with pure water due to their hydrophilic nature. Thus, Figure 5 suggests that one shall choose mixtures of alcohol and water (50% E-50% W), rather than pure water because larger phenolic amounts and lower amount of carbohydrates are extracted at the same time. Figure 5 also shows that temperature increase at values more than 50 °C resulted in the extraction of more carbohydrates, for both solvents, while in the case of phenolics, the temperature effect is significant only for the mixture of ethanol and water. For the case of extraction of phenolics using pure water, Figure 5 shows that initially there is a decrease in TPC values in the range of 10–20 °C whereas above 20 °C a slight increase in TPC values was observed. Comparing TPC and carbohydrates recovery, it seems that the optimum temperature range is between 30 and 50 °C. However, since the TPC concentration values are enough for temperature values above 25 °C, this temperature was adopted as optimum for practical reasons and for energy economy. Most of the reported literature data [34,35,36,37,38,39,40] suggest extraction at ambient temperature because when temperature is high, the extraction rate is not enhanced, and the process becomes more expensive. Extreme large temperature conditions (superheated as in [39]) are undesirable in order to avoid possible phenolic decomposition.

#### 3.1.5. Optimization of the Maximum Recovery of Phenolics from the Solid Waste

**Figure 6:**
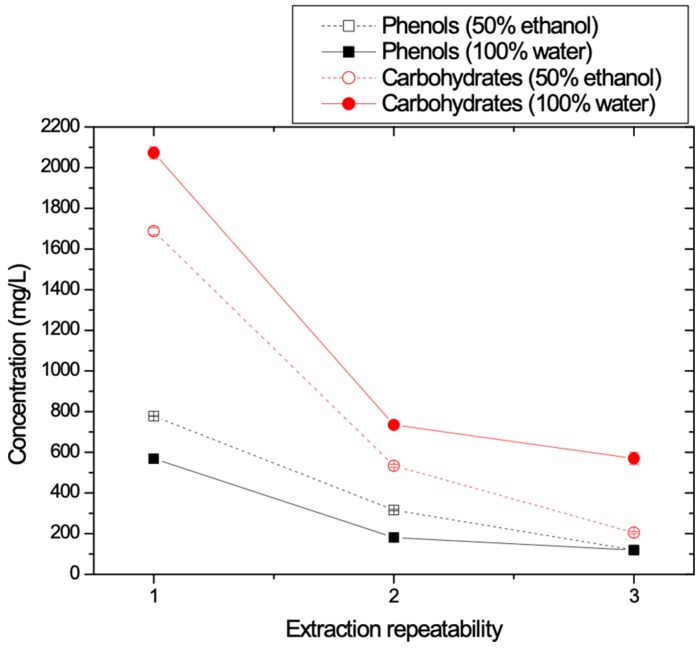
TPC and Carbohydrates concentration values versus repeatability with respect to the solid residue.

At this stage, three successive extraction experiments were performed using the same quantity of 40 g of solid waste and fresh solvent each time. The experiments were conducted at 25 °C, under stirring (100 rpm) for 1 h for both tested solvents (pure water and 50% E-50% W). In Figure 6 the obtained values for TPC and carbohydrates are presented as function of extraction repeatability. It is shown that the second and the third trials for recovery of phenolics and carbohydrates from the same solid waste resulted in much reduced recovery rates. It is clear that after the third cycle all material available for extraction was removed from the solid matrix. The mixed solvent (50% E-50% W) seems to enhance phenolics recovery whereas carbohydrates recovery is reduced. Thus, it is concluded that TPC and carbohydrates recovery are high enough from the first extraction and no significant increase is obtained during the second and third extraction.

#### 3.1.6. Effect of Stirring Rate

**Figure 7:**
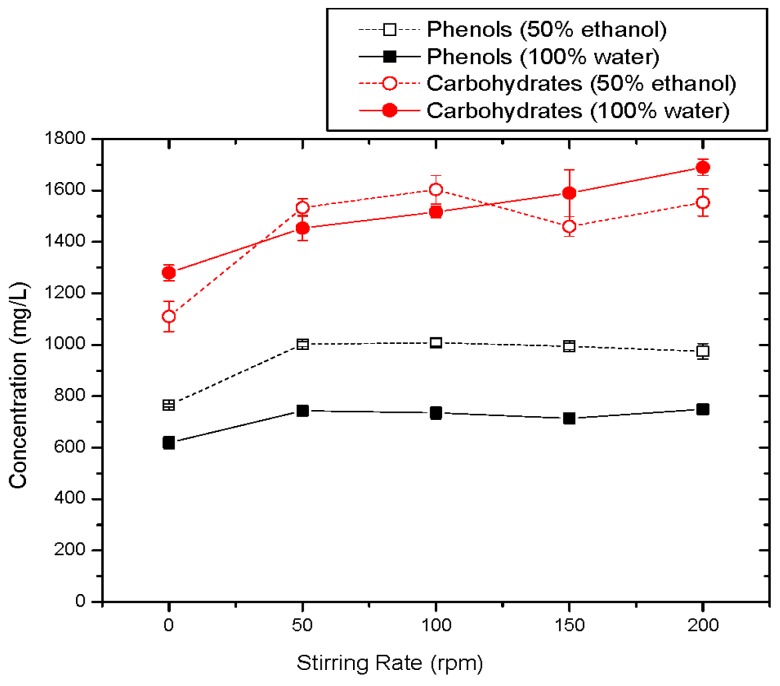
TPC and carbohydrates concentration values versus stirring rate.

The effect of stirring on TPC and carbohydrates recovery during the extraction procedure is depicted in Figure 7. Concerning the TPC concentration values (solid and dot black curves), an increase is observed up to 50 rpm for both solvents. By further increasing the stirring rate up to 150 rpm, no significant change in the phenolic load was observed. A slight extra increase is obtained at 200 rpm in the case of pure water. Carbohydrates concentration values obtained during extractions with water solvent were increased with increasing stirring rate. When the used solvent is 50% E-50% W, carbohydrates concentration values were increased up to 100 rpm, then a slight decrease was obtained at 150 rpm which was followed by an increase at 200 rpm. Comparing TPC and carbohydrates concentration values, the optimal stirring rate is found at 100 rpm since at this rate TPC recovery was maximized. Stirring rate at 100–200 rpm of 1–2 h is acknowledged in most the reported works.

#### 3.1.7. Effect of the Extraction Time

**Figure 8:**
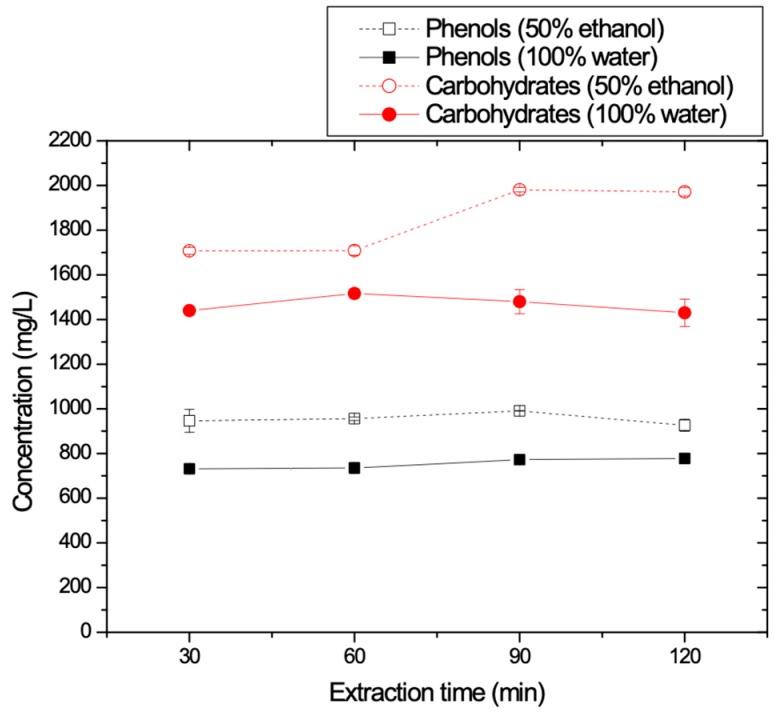
TPC and carbohydrates concentrations as function of the duration of extraction.

At this stage, the time for the total duration of the extraction was investigated. As previously, 40 g of olive oil mill waste were extracted using each time 100 mL of the two different solvents, at 25 °C and 100 rpm of stirring rate. TPC and carbohydrates concentrations for the different types of solvents and for increasing extraction time are depicted in Figure 8. It is shown that for both solvents, after 60 min of extraction there is no significant change in the TPC concentrations. Regarding carbohydrates concentration (red curves), the concentrations remain constant for the first hour of extraction for both solvents. For longer extraction durations, the carbohydrates recovery is reduced when the solvent is pure water, whereas it was increased significantly when the solvent used consisted of 50% E-50% W. Thus, it is concluded that the optimum extraction time is 1 h (60 min) since TPC concentration remains constant after 1 h and at the same time the recovery of carbohydrates was not too high.

The optimal conditions for the present experimental analysis are referred to the maximum recovery of phenolic substances and if possible, to the minimum recovery of carbohydrates. From the above analysis of all experimental data, for both solvents (pure water and 50% E-50% W), the optimum TPC extracted values were achieved using 40 g of two-phase olive oil mill waste, 100 mL of solvent, without the addition of HCl, at room temperature 25 °C, after 1 h stirring at 100 rpm.

The difference between the two types of solvents lies in the recovery of the phenolic substances which in the case of 50% ethanol in water is higher (Table 1). When 50% of ethanol in water is used as solvent, the maximum recovery in phenols is about 1g (mean value 970 mg) TPC per L of two-phase mill waste extract, whereas when pure water is used as solvent, a significantly lower amount of TPC (~660 mg TPC/L of waste) is obtained. This difference is attributed to the higher solubility of polyphenols in organic solvents than in water. Although the parametric investigation concerns mainly the higher TPC recovery, it is important to investigate also the optimum recovery of total carbohydrates. As Table 1 shows, the obtained carbohydrates concentration values are similar for both solvents, thus, the choice of solvent used in the extraction process does not affect the recovery of total carbohydrates.

### 3.2. Pilot scale Experiments

Pilot scale experiments for the treatment of two-phase olive mill wastes (pomace) or for the treatment of olive mill wastewaters show an excellent performance if a successful pretreatment step is used before the process of membrane filtration [2,8,15,18,20,21,22,24,27,28,40]. The plethora of works on the implementation of a combination of different membranes in a raw, allow researchers to further suggest the exploitation of membranes concentrates in order organic compounds (such as the phenolics to isolated). Lafka et al. [40] stated that a combination of acidification, fat removal, and a two-step extraction with ethanol can be applied in larger volumes of pomace. Sabatini [41] reported new technologies from the recovery of phenolics from olive oil and olive mill wastewaters. Agalias et al. [42] developed a 3-stage purification system to isolate phenolic compounds (filtration, adsorption/desorption on specific resins and purification of hydroxytyrosol via chromatography. Zagklis et al. [29] suggested a scheme of UF, NF and RO followed by adsorption/desorption on specific resins to isolate phenolics. Pizzichini and Russo [43] submitted a patent for the recovering components of OMW with membrane technologies.

**Figure 9:**
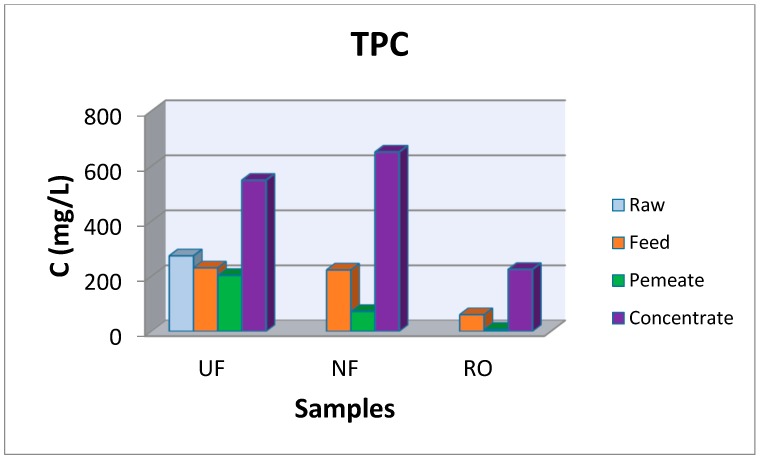
TPC concentration at the raw, feed, permeate and concentrate streams at the UF, NF, and RO membranes.

**Figure 10:**
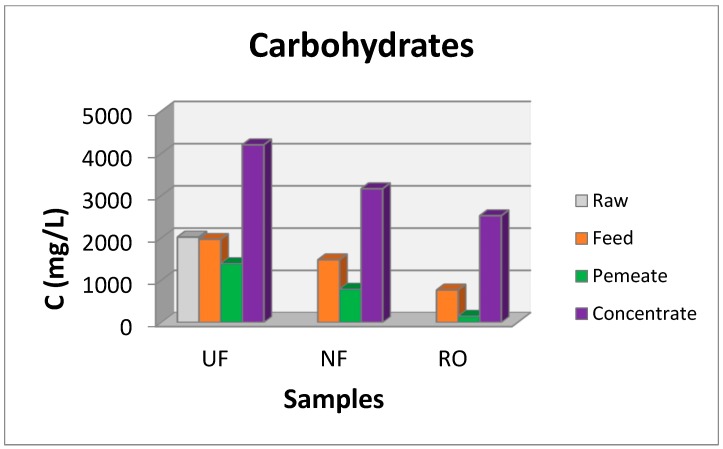
Carbohydrates concentration at the raw, feed, permeate, and concentrate streams at the UF, NF, and RO membranes.

**Figure 11:**
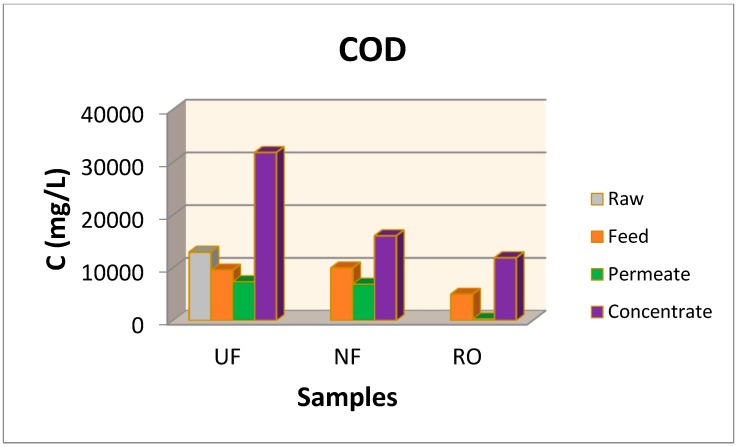
Chemical oxygen demand at the raw, feed, permeate and concentrate streams at the UF, NF, RO membranes.

In order to investigate the possibility of TPC recovery from larger volumes of waste, pilot scale experiments were performed using the pilot plant of UF, NF, and RO membranes described previously (described also in details in [15,21,29]). Although during the parametric study, the optimum ratio (waste mass to solution volume) was found to be 1:5, a smaller ratio (1:20 ratio) was chosen in order to avoid membranes fouling. Additionally, although the solvent 50% E-50% W was found as optimum, pure water was chosen for the extraction of organics from the OWSM in pilot scale experiments in order to reduce the experimental costs. TPC and carbohydrates concentrations as well as COD values measured at all stages of the experiment in pilot scale at all fractions of the concentrate and permeate streams are summarized in Figure 9, Figure 10 and Figure 11. In a previous work [15], salinity was found between 0.22–0.26% in OMW samples whereas it was found equal to zero in the final RO permeate.

According to the experimental data obtained for the TPC (Figure 9), a considerable amount of TPC is recovered at the concentrate stream of the UF reaching ~550mg/L and at the concentrate stream of NF membrane, where 652 mg/L were retained. In the concentrate stream of RO, 225 mg/L of phenolics were isolated and it is assumed that this fraction contains simple phenols with low molecular weight size. The corresponding values for the permeate stream for the three membranes were 203, 37, and 7 mg/L for UF, NF and RO streams, respectively. TPC concentration at the final effluent (permeate after the RO membrane) is less than 10 mg/L, showing that the polyphenols of the olive oil mill waste were almost completely recovered in the concentrate streams. Thus, the final stream can be disposed for irrigation purposes of cultivated fields or fruit trees. All selected streams are treated further to remove most of the solvent in order phenolic samples with high purity to be isolated. In Zagklis et al. [29] it is shown that samples with phenolic compounds up to 38% can be isolated if the suggested with adsorption/desorption using resins is involved. Agalias et al. [42] following the three-stage purification system, succeeded in obtaining hydroxytyrosol solutions of high purity (80%).

Carbohydrate recovery showed a similar behavior as the recovery of phenols, when the extracted sample was filtered in the proposed scheme of UF, NF, and RO modules. Carbohydrates concentration values (Figure 10) show a decrease at the concentrate stream after NF and RO modules, as expected, since polysaccharides are large molecules and are retained in the concentrate stream of UF. The carbohydrates concentration at the concentrate streams of the UF module was ~4200 mg/L, at the NF module was ~3200 mg/L and at the RO module was 2500 mg/L. Carbohydrates concentration at the final effluent (permeate stream of RO) is small (~146 mg/L), whereas at the intermediate steps of the UF and NF permeate stream, concentration values were 1780 mg/L and 787 mg/L, respectively. The concentration of carbohydrates at the final effluent at 146 mg/L was found to be more than the accepted limits for free disposal to aqueous receptors, however, it is small and safe enough for irrigation purposes. The presence of carbohydrates in the same solution with the phenolic compounds was reported by many researchers [29,34,35,36,37,38,39,40,41,42] and extra steps shall be adopted for further purification of phenolic compounds using techniques as the adsorption/desorption on specific resins [29,42].

Figure 11 shows the corresponding COD values obtained in the concentrate and permeate streams of the membrane scheme. The highest COD concentration was obtained after the implementation of UF membrane at its concentrate stream as it exceeded the 32,000 mg/L.

The concentrate stream of NF contained 16,000 mg/L of organics expressed as COD values and at the concentrate step of RO, the retained organics had a concentration of 12,000 mg/L. The COD at permeate stream of UF, was up to 7360 mg/L, at NF was 6885 mg/L and after the RO membrane was 284 mg/L. This final value for COD is too low in comparison with the values at the feed of the membrane module showing that the final permeate is almost clean. Similar results for the reduction of COD values (more than 80 %) was observed in most of the works reported here [2,3,8,15,18,20,21,22,28,29,41,42,43].

## 4. Conclusions

Extraction experiments were performed in order to investigate the optimal conditions for the isolation of the maximum content of phenolic compounds and if possible, the minimum extraction of carbohydrates. Two different types of solvents were examined (pure water, ethanol, and mixtures of ethanol and water) in series of experiments where the effect of different parameters was investigated, i.e., maximum solid to solvent ratio, the addition of HCl, the temperature during the extraction (10–60 °C), the extraction repeatability, the rate of stirring (rpm) and the extraction time. When the solvent of 50% E-50% W was used, TPC and carbohydrates concentrations were found to increase in all tests. Extraction repeatability on the same waste using new solvent each time, was found to slightly increase TPC recovery. Increasing the stirring rate increases the carbohydrates concentration but in the case of TPC recovery, it is almost stabilized after 100 rpm. The optimal TPC recovery was achieved for both solvents after the extraction of 40 g of OWSM, without the addition of HCl, at room temperature 25 °C, after 1 h stirring at 100 rpm.

Finally, a pilot scale membrane set-up consisting of UF, NF, and RO membranes was used for the recovery of TPC and carbohydrates. The TPC and carbohydrates concentration, as well as the COD values were measured at the raw olive oil mill waste, at the filtered feed stream of the UF, and at all permeate and concentrate streams, after each membrane unit. The final effluent in the permeate stream after the RO membrane was found almost clean from polyphenols, whereas low concentration of carbohydrates and low COD values were detected. TPC at the concentrate stream of RO was 225 mg/L of phenolics whereas at the permeate was lower than 10 mg/L. Carbohydrates concentration at the final effluent was found equal to 146 mg/L and COD was almost ~284 mg/L. These concentration values give the evidence that it is possible to clean the two-phase olive mill solid waste (OMSW-pomace) with simultaneous recovery of high amounts of phenolic substances. The high recovery of polyphenols is of significant interest due to their high added value whereas at the same time the clean final permeate can be reused in other applications.

## Figures and Tables

**Table 1:** TPC and carbohydrates concentration values (mg/L) in the extracted solution.

Parameter	Maximum TPC Concentration (mg/L)		Maximum Carbohydrate Concentration (mg/L)	
Solvent Type Conditions	100% H_2_O	50% E (95%), 50%W	100% H_2_O	50% E (95%), 50% W
Temperature, 20 °C	471.0	1020.3	2060.0	1373.3
Rate, 100 rpm	735.5	1008.3	1516.7	1603.3
Duration, 60 min	735.5	956.3	1516.7	1708.3
HCl, Ml	678.0	892.5	1436.0	1605.3
Average Concentration	655.0	969.3	1632.3	1572.6
